# A pre-post pilot feasibility study of the MindBia web-based nutrition intervention for individuals with severe mental illness: Study protocol

**DOI:** 10.1371/journal.pdig.0001756

**Published:** 2026-09-25

**Authors:** Ciara O’Sullivan, Tara Coppinger, Indika Dhanapala, Diarmuid Boyle, Alison Merrotsy

**Affiliations:** 1 Department of Sport, Leisure & Childhood Studies, Munster Technological University, Bishopstown, Cork, Ireland; 2 Nimbus Research Centre, Munster Technological University, Bishopstown, Cork, Ireland; 3 Health Service Executive (HSE), Kerry, Ireland; 4 Department of Applied Social Studies, Munster Technological University, Bishopstown, Cork, Ireland; Yonsei University College of Medicine, KOREA, REPUBLIC OF

## Abstract

People living with severe mental illness experience substantial health inequalities and high rates of premature mortality, largely driven by modifiable lifestyle-related risk factors, including poor diet quality. Digital technologies may provide scalable and flexible opportunities to support healthier behaviours; however, few interventions have specifically targeted nutrition-related outcomes as a primary focus in this population. MindBia is a co-designed, web-based digital nutrition intervention consisting of six structured modules developed for individuals residing in high-support mental health hostels. The intervention was informed by prior studies with service users and mental health staff. This pilot study aims to evaluate the feasibility, acceptability, and preliminary effectiveness of the MindBia intervention over eight weeks. A mixed-method pre-post design will be employed. Participants will complete baseline and post-intervention assessments examining dietary behaviours, digital literacy, and general wellbeing. Engagement with the web-based application will be monitored using embedded usage analytics. Semi-structured interviews conducted post-intervention will explore participants’ experiences, perceived impact, and barriers to engagement. Findings from this pilot study will inform refinement of the MindBia web-based application and guide the design of a larger scale trial evaluating its overall effectiveness.

## Author summary

Severe mental illness is associated with significant physical health inequalities and reduced life expectancy. Poor diet is a major contributing factor, yet structured nutrition support tailored to this population remains limited. Many digital interventions address physical activity or smoking for people living with severe mental illness, but few focus specifically on nutrition-related outcomes, particularly those residing in high-support mental health hostels. MindBia was developed as a web-based nutrition intervention co-designed with individuals with severe mental illness and mental health staff. The intervention consists of six structured modules designed to improve food knowledge, practical skills, and confidence using accessible digital technology. In this pilot study, we are evaluating whether delivering MindBia is feasible, acceptable, and preliminary effective. By examining engagement and user experiences, we aim to refine the intervention and inform the design of a larger trial to evaluate its overall effectiveness. Accessible, co-designed digital nutrition interventions may represent an important step toward reducing health inequalities in this underserved group.

## 1 Introduction

People living with severe mental illness (SMI) experience some of the most profound health inequalities globally, with these disparities well documented in the literature [1,2]. These inequalities remain a leading contributor to global disease burden and are associated with significantly elevated rates of premature mortality [3]. Modifiable lifestyle-related risk factors - including suboptimal diet quality, unhealthy eating behaviours, high smoking prevalence, low physical activity, inadequate sleep, and high levels of sedentary behaviours are highly prevalent within this population [4]. These behaviours contribute to increased physical comorbidities, can exacerbate mental health conditions [4] and play a central role in adverse physical and mental health outcomes [5]. Addressing these lifestyle behaviours through intervention, therefore, represents a critical opportunity for reducing the mortality gap experienced by individuals with SMI [6].

Digital technology presents a promising avenue for transforming mental health care delivery at scale [5]. A diverse range of digital tools are increasingly integrated in mental health care [7–9]. Electronic health (eHealth) refers to the use of information and communication technologies (ICT) to support health care delivery [10]. Mobile health (mHealth), a subset of eHealth, and web-based platforms can deliver cost-effective, scalable, and flexible interventions that support healthier lifestyle behaviours [11,12]. These technologies create opportunities to provide personalised and accessible health information, enhance digital and health literacy, deliver tailored support for behaviour change, and enable continuous monitoring to facilitate sustained lifestyle improvements [13].

Recent evidence has begun to examine digital lifestyle interventions for individuals with SMI

A systematic review [14] that evaluated digital health behaviour change interventions in this population found most studies target smoking cessation or physical activity. While interventions were generally acceptable and feasible, evidence for improvements on mental health outcomes was limited [14]. Notably, none of the included studies specifically addressed nutrition-related outcomes. More recently, Holmes et al. (2025) conducted a systematic review of 11 eHealth- delivered dietary interventions in adults with SMI, reporting positive effects on anthropometric outcomes, physical activity, mental health, and cardiovascular measures. However, diet itself was assessed in only a few studies (N = 3).

Previous research conducted by the authors [15] suggests that digital technology may offer a promising support model for supporting poor dietary behaviour, with the potential to address many of the structural and attitudinal barriers experienced by the SMI population. However, challenges such as digital exclusion and low digital literacy persist [15,16]. Incorporating public and patient involvement (PPI) alongside behavioural science frameworks during intervention development may enhance their relevance, acceptability, and potential impact [15].

To address this gap, ***MindBia*** was developed as a co-designed, web-based digital nutrition intervention tailored for individuals with SMI residing in high-support mental health hostels. The intervention development process followed a structured, theory-informed, multi-phase approach guided by the Behaviour Change Wheel (BCW) [17,18].

This study represents the third and final stage of the research, comprising:

- **Study 1.** Behaviour analysis of adult nutrition behaviours in Irish Mental Health High Support Hostels according to the COM-B model [19].
- **Study 2**. Co-design of the MindBia digital intervention with service users and mental health staff, guided by the BCW and co-design principles [20].
- **Study 3.** A pre-post pilot feasibility study to evaluate the MindBia web-based intervention (current study).

The primary aim of this pilot study is to investigate the feasibility and acceptability of delivering the MindBia web-based application to individuals with SMI living in Irish high-support mental health hostels. A secondary aim is to explore its preliminary effects on nutrition-related behaviours, digital literacy, and wellbeing outcomes.

## 2 Materials and methods

### 2.1 *Ethics statement*

Full ethical approval was granted from Clinical Research Ethics Committee (CREC) on the 5^th^ of June 2026. CREC Review Reference Number: ECM 4 (m) 03/03/2026 & ECM 5 [5] 03/03/2026 & ECM 3 (n) 14/04/2026. All participants will provide written informed consent prior to participation. Data will be pseudonymised and stored securely in accordance with institutional data protection policies. Participants may withdraw from the study at any time prior to data pseudo-anonymisation without consequence.

### 2.2 *Study design: The MindBia web-based intervention*

This protocol describes an eight-week mixed-method, pre-post feasibility study of the MindBia web-based nutrition intervention. The study will incorporate quantitative outcome measures, app usage analytics, and post-intervention qualitative interviews to evaluate the implementation and delivery of the intervention.

### 2.3 *Setting*

The study will be conducted in four high-support mental health hostels located in the south-west of Ireland. These residential settings provide accommodation and 24-hour structured support for adults with severe mental illness. Daily support is provided by mental health nurses, healthcare assistants, and household staff, with clinical oversight provided by a consultant psychiatrist and dedicated doctor across the four hostels. The participating hostels vary in size, with capacity ranging from 8 to 14 residents.

### 2.4 *Participants*

#### 2.4.1 *Recruitment.*

Participants will be recruited in collaboration with staff from high-support mental health hostels. As this is a pilot feasibility study, a formal sample size calculation was not undertaken. Consistent with recommendations for feasibility studies, the sample size was pragmatically based on the available study population [21]. All eligible residents across the participating hostels (N = 43) will be invited to participate through information sessions, study information leaflets, and staff referral. Individuals expressing interest will be screened for eligibility in consultation with the relevant clinician and provided with detailed study information. Written informed consent will be obtained prior to participation. Capacity to provide informed consent will be determined by the relevant clinician prior to study enrolment. Residents experiencing an acute episode at the time of recruitment may be temporarily deferred from participation. They will be offered the opportunity to join or re-join the study once their clinician confirms they are clinically well enough to participate.

#### 2.4.2 *Inclusion criteria.*

Participants will be eligible for inclusion if they are aged 18 years or older, have a clinical diagnosis of severe mental illness, are current residents of a high-support mental health hostel, and have the capability to provide informed consent. Participants will be excluded if they have severe cognitive impairment that would prevent independent engagement with the web-based application, or if they have insufficient English literacy to engage with the intervention content.

### 2.5 *Study duration and procedures*

The study will be conducted over an eight-week period and will consist of the following phases:

- **Week 1 (Baseline):** Collection of demographic and clinical information and administration of baseline quantitative measures (MEDAS [22], Self-Reported Questionnaire, Technology Use Survey [23]; WHO-5 [24]).
- **Week 2–7 (Intervention):** Participants will engage with the MindBia web-based application, completing one module per week (six modules in total). Weekly check-ins will be conducted by the research team to encourage adherence and address any technical or content-related issues. App engagement will be monitored continuously via embedded analytics. No additional outcome data will be collected during weekly check-ins.
- **Week 8 (Post-intervention):** Participants will complete post-intervention quantitative measures (MEDAS [22], Self-Reported Questionnaire, Technology Use Survey [23], WHO-5 [24]) and participate in semi-structured interviews to explore their experience, perceived impact, and feasibility of implementation.

#### 2.5.1 *Materials.*

Each hostel will be provided with one iPad preloaded with the MindBia Application. Supplementary printed materials will also be made available, where appropriate to support engagement. These materials will include a nutrition booklet summarising key messages from the modules, visual prompts such as posters or graphics displayed in the service user kitchen, and practical aids such as Safefood portion cups to support food preparation and portion awareness.

### 2.6 *Intervention development*

#### 2.6.1 *Phase 1. Behaviour analysis* [19].

Phase 1 has been completed and examined nutritional behaviours, barriers, and facilitators to nutrition behaviours and digital technology use among individuals with SMI. A mixed-method design was employed, incorporating both quantitative (questionnaire) and qualitative (observation) approaches. The study was conducted across four high-support mental health hostels and included nineteen staff/clinicians (10 females, 9 males; aged 18–64 years), and 33 service users (19 females, 14 males; aged 30–90 years). Participation was voluntary, and written informed consent was obtained from all participants. The questionnaire assessed staff/clinicians’ perceptions of service users’ nutrition behaviours and digital technology capabilities, opportunities and motivations (COM-B model) [17].

Observations captured food choices, eating behaviours, and fluid intake. Findings indicated that facilitators included psychological and physical capability to engage in nutrition-related activities, while barriers included limited food autonomy, lack of fruit and vegetables, and inconsistent access to water. Technology use was limited, with lack of access and low digital literacy identified as key barriers. Findings suggest that digital nutrition interventions could be effective if they first address barriers such as digital literacy and/or meal preparation skills and are co-designed with service users to ensure digital tools are user-friendly and engaging.

#### 2.6.2 *Phase 2. Co-design of the MindBia application* [20].

Phase 2 has been completed and involved a structured co-design process with service users and mental health staff (distinct from those involved in Phase 1) to support the development of the “MindBia” web-based application. This phase built directly on findings from Phase 1 and was guided by co-design principles and behavioural science frameworks [17,18,25]. The full protocol for this phase has been published previously [20]. The MindBia web-based application was co-designed through a structured, multi-stage co-design process involving 20 participants (13 females, 7 males). Co-design activities were conducted to ensure that the proposed intervention content, format, and delivery would be acceptable, relevant, and appropriate for individuals with SMI. A combination of individual interviews (N = 11) and an in-person focus group (N = 9) was used and participants were based across Ireland and England, with ages ranging from 18-66 years.

#### 2.6.3 *Integration of findings.*

Findings from both Study 1 (behaviour analysis) and Study 2 (co-design study) have directly informed:

- Selection of target behaviours
- Mapping of COM-B components
- Selection of Behaviour Change Techniques (BCTs)
- Tailoring and accessibility of content
- App structure and usability

Subsequently, based on the evidence from these earlier phases, a six-module digital nutrition intervention has been developed (Table 1). BCTs are reported using the Behaviour Change Technique Taxonomy [26]. BCTs codes are presented numerically (e.g., 4.1 = Instruction how to perform a behaviour; 6.1 = Demonstration of the behaviour).

**Table 1:** Overview of modules, key messages, target behaviours, behaviour change techniques (BCTs), mode of delivery, COM-B components and intervention functions.

Module	Key Messages	Target Behaviour	Behaviour Change Techniques (Behaviour Change Technique Taxonomy)	Mode of delivery (Behaviour Change Technique Taxonomy)	COM-B Component/s	Intervention Functions (Behaviour Change Technique Taxonomy)
**1. Introduction**	• Intro to app, features, how to use, basic internet navigation • Elements from Digital Outreach for Obtaining Resources and Skills (DOORs)	Digital literacy, app navigation	Instruction on how to perform a behaviour (4.1) Demonstration on behaviour (6.1) Behavioural practice/rehearsal (8.1) Social support (3.1)	In-person demo (4.1;6.1) In-app demo (4.1;6.1) Interactive tasks (8.1)	Physical Capability	Training (4.1;6.1;8.1) Enablement (3.1)
**2. Diet and Mental Health**	• Importance of diet for mental health • Connections between food and mood • Mediterranean diet principles • Impact of medication	Knowledge and awareness about diet-mood link, mediterranean principles, and medication effects	Information about health consequences (5.1) Information about social/environmental consequences (5.3) Behavioural practice/rehearsal (8.1) Credible source (9.1)	Short videos (5.1) Infographics/fact sheets (5.1;5.3) Interactive quiz (8.1)	Psychological Capability; Reflective Motivation; Physical Capability	Education (5.1) Persuasion (5.3) Training (8.1)
**3. Food Groups**	• Irish Food pyramid **•** Breakdown/explanation of food categories (fruit, veg, protein, etc)	Understand Irish food pyramid categories	Information about health consequences (5.1) Behavioural practice/rehearsal (8.1)	Intro on food groups (5.1) Visual portion sizes (5.1) Meal building game (8.1) Meal checklists (8.1)	Psychological Capability	Education (5.1) Training (8.1)
**4. Meal Preparation Skills**	• Cooking can be simple and fun • Simple cooking skills • Meal preparation tips for low-energy days **•** Information about different utensils	Encourage cooking, practical skills	Behavioural practice/rehearsal (8.1) Demonstration of behaviour (6.1)	Healthy recipes (8.1) Simple instructions (6.1) Short videos (6.1)	Physical Capability; Physical Opportunity	Education (8.1) Training (6.1)
**5. Shopping**	• Budget friendly shopping tips **•** Making healthy choices on a budget	Budgeting, making healthy choices	Behavioural practice/rehearsal (8.1) Action planning (4.1)	Interactive tips (8.1) Budget strategies (4.1)	Physical Capability; Psychological Capability	Training (8.1) Enablement (1.4)
**6. Hydration**	• Importance of water intake • Sugar content of other drinks	Learn importance of water	Information about health consequences (5.1) Behavioural practice/rehearsal (8.1)	Infographics (5.1) Quizzes (8.1)	Psychological Capability; Physical Capability	Education (5.1) Enablement (8.1)

Additional behaviour change techniques and implementation components supporting the intervention are presented in Table 2.

**Table 2:** Supplementary behaviour change techniques (BCTs), intervention functions, and delivery modes.

COM-B Component/s	Intervention Functions	BCTs	Mode of delivery
**Physical Opportunity**	Environmental Restructuring	Adding objects to the environment (12.5)	iPads to hostels Nutrition booklet Add posters/graphics to walls in service user kitchen (visual prompts or tips) booklet, safe portion cups
**Psychological Capability**	Enablement	Goal setting (1.1)	Weekly goals per module
**Reflective Motivation**	Incentivisation	Feedback on behaviour (2.2)	Digital badges awarded per module completion Progress Dashboard End of module messages *(congrats you’ve completed this module!”)* End of intervention shopping voucher intended to support the purchase of healthy food items , “CONGRATS YOU’VE COMPLETED ALL MODULES YOU HAVE EARNED A *30 EURO FOOD VOUCHER”

### 2.7 *Outcome measures*

#### 2.7.1 *Participant characteristics.*

Demographic and clinical information will be obtained via clinicians working within participating high-support mental health hostels. Service users will not be required to provide this information. Only the following data will be recorded: age, sex, gender, and ethnicity; current mental health diagnosis or diagnoses; and current length of stay within the hostel.

#### 2.7.2 *Primary outcomes.*

*2.7.2.1 Feasibility.*

Feasibility will be evaluated through recruitment, retention, intervention adherence (module completion), and participant engagement with the MindBia web-based application. Recruitment and retention rates will be recorded throughout the study. Intervention adherence and engagement will be assessed using embedded app analytics collected continuously during the intervention period. No personal or identifiable data will be collected by the application. Usage metrics will include total number of logins, time spent within each module, modules completed, and week-by-week engagement.

*2.7.2.2 Acceptability.*

Acceptability will be explored through post-intervention semi-structured interviews (week 8) (S1 Text) conducted with a purposive sample of participants. Interviews will explore participants’ experiences using the MindBia application, perceived changes in nutrition-related behaviours and digital literacy, and views on feasibility, acceptability, and sustainability. The qualitative component is informed by an interpretivist perspective, recognising that participants’ experiences and perceptions of the intervention may be shaped by their individual contexts. Interview guides will be piloted with co-design participants and refined accordingly. To support engagement and facilitate discussion, a range of interactive and visual prompts may be used where appropriate (e.g., images or prompt cards illustrating food groups, shopping choices, and hydration behaviours, such as food plates, shopping baskets for water glass icons).

#### 2.7.3 Secondary outcomes.

The secondary outcomes will be measured at baseline (week 1) and post-intervention (week 8).

*2.7.3.1 Nutrition-related outcomes.*

Overall dietary pattern change will be assessed using the Mediterranean Diet Adherence Scale (MEDAS) [22] (S2 Text). Although originally developed to assess adherence to a Mediterranean dietary pattern, MEDAS provides a brief, validated tool to capture general shifts in overall healthy eating behaviours.

As MEDAS does not capture all behaviours targeted by the MindBia intervention (meal preparation, food shopping, hydration) a small number of module specific self-report items will be added and used to assess pre- and post-intervention changes in key behaviours (S3 Text). These items have been designed to align with the MindBia intervention content and the Irish Food Pyramid and will be piloted with co-design participants prior to data collection. Visual aids (e.g., printed Irish Food Pyramid, portion size images, measuring cups) will be available during questionnaire completion to reduce cognitive burden. Terminology of MEDAS will be adapted for the Irish context. Qualitative interviews conducted post-intervention (week 8) will further explore perceived dietary change and contextual factors influencing behaviour change.

*2.7.3.2 Digital literacy and technology use.*

Digital access and technology use will be assessed using an adapted Technology Use Survey [23] (S4 Text). This instrument was initially developed to evaluate individuals’ ability to use and interact with digital technologies and identify barriers to digital engagement. It has previously been piloted across diverse settings, including inpatient psychiatry units and community mental health services [23]. For the present study, the survey will be adapted to align with the MindBia intervention and administered at baseline (week 1) and post- intervention (week 8). Perceived changes in digital skills and confidence, barriers and facilitators of technology use, and experiences of using the MindBia application will be explored further through qualitative interviews conducted post-intervention (week 8).

*2.7.3.3 General wellbeing.*

Subjective wellbeing will be assessed using the World Health Organisation – Five Well-being Index (WHO-5) (S1 Fig) [24]. The WHO-5 is a brief, five-item measure of current mental wellbeing and has demonstrated adequate validity both as a screening tool for depression and as an outcome measure in clinical trials across a wide range of clinical and general populations [24].

In this feasibility study, the WHO-5 will be administered pre- and post-intervention as a secondary, exploratory outcome to assess any potential changes in wellbeing following engagement with the MindBia intervention.

### 2.8 *Statistical analysis*

Descriptive statistics will be used to summarise baseline characteristics (age, gender, mental health diagnosis), and app usage patterns, including frequency and duration of use, and module completion rates. Pre- and post-intervention comparisons will be conducted to explore key changes in outcomes; dietary change (MEDAS; self-reported questionnaire), general wellbeing (WHO-5), and digital literacy (Technology Use Survey). Changes will be assessed by comparing scores before and after the intervention, and by using appropriate paired statistical tests (e.g., Paired sample t-tests or Wilcoxon signed-rank test). Missing data will be reported descriptively, and analyses will be conducted using available data only. Effect sizes will be calculated, where appropriate, to estimate the magnitude of any observed changes.

Given the pilot nature of the study, analyses will be exploratory and not powered to detect definitive effectiveness. Qualitative interviews will be audio-recorded, transcribed verbatim and analysed using reflexive thematic analysis [27]. Quantitative and qualitative findings will be integrated at the interpretation stage to provide a comprehensive understanding of the feasibility, acceptability, and preliminary outcomes of the MindBia intervention.

### 2.9 *Potential risks*

The study is considered to be minimal risk; however, participants may experience frustration or uncertainty when using the digital device or navigating the MindBia application. To mitigate this, all efforts will be made to ensure the participants feel confident using the iPad and accessing the intervention content. Brief, supportive check-ins by the research team will be available to address any technical issues or concerns.

All study procedures will be conducted in collaboration with hostel staff. Participants will complete the questionnaires and interviews with the researcher, while engagement with the MindBia application will occur independently during the intervention period. A clinician or appropriate hostel staff will be available within the setting if required. There is minimal risk that completing the questions or participating in interviews about diet, wellbeing, or technology use could cause mild discomfort. Participants will be informed that they may skip any question they do not wish to answer and may withdraw from participation at any time. If a participant becomes distressed during any research activity, the researcher will pause the activity and refer the participant to appropriate hostel staff member or clinician for support.

Participants who experience an acute episode during the intervention period will be supported by their clinical care team. Where appropriate, they may be offered the opportunity to re-engage with the intervention stage without penalty. However, depending on duration of absence and timing within the study period, their data may not be included in the final analysis. Participants will also be provided with additional information or resources related to nutrition, wellbeing, or digital technology if requested.

**Fig 1:**
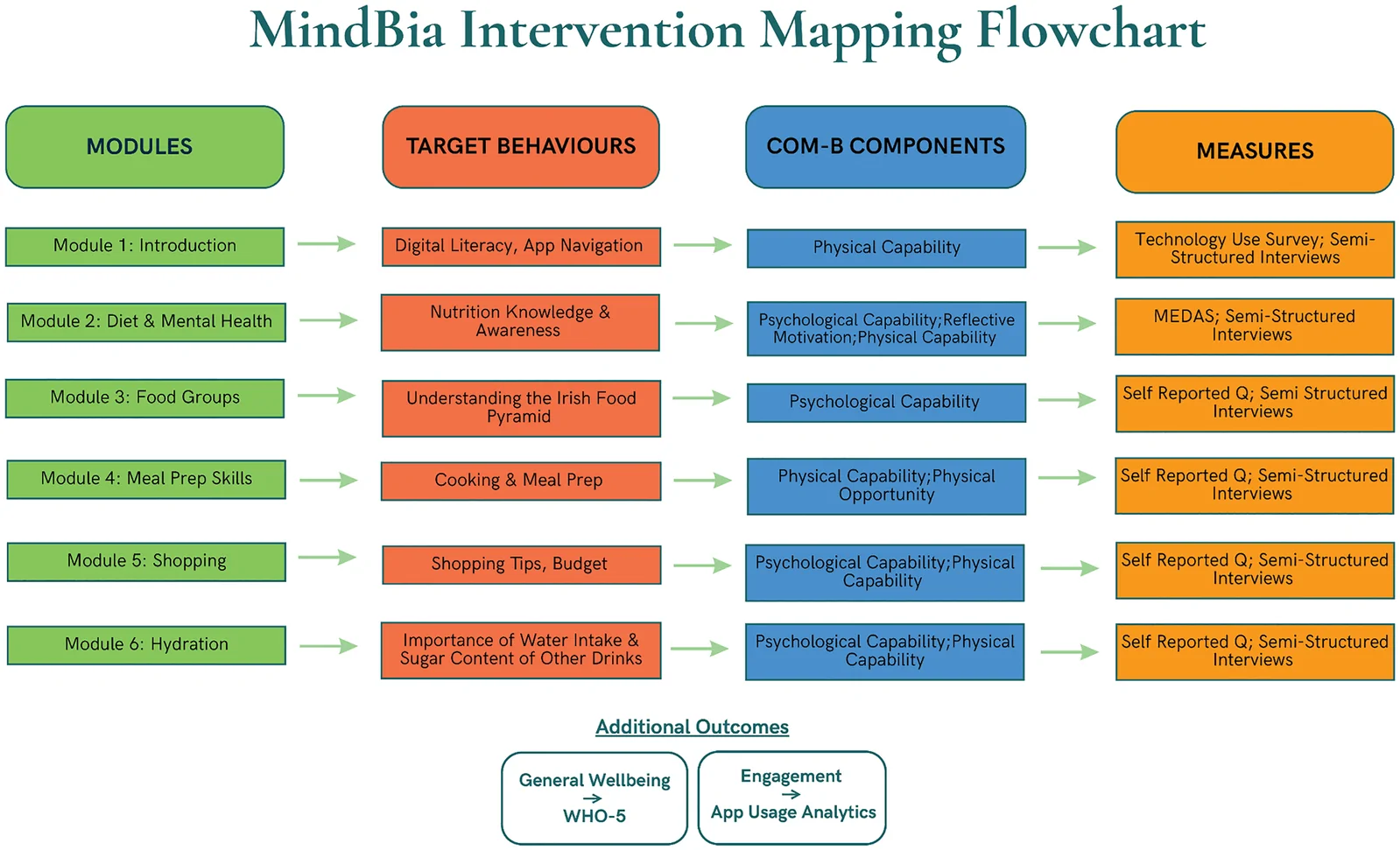
MindBia intervention mapping.

Fig 1 illustrates the MindBia intervention mapping, showing the progression from intervention modules, their linked target behaviours and COM-B components, and corresponding outcome measures.

## 3 Discussion

The MindBia pilot study has been designed to explore the feasibility, acceptability, and preliminary effectiveness of a co-designed, web-based digital nutrition intervention for individuals with SMI residing in Irish high-support mental health hostels. The intervention leverages digital technology to support healthier nutrition behaviours, enhance digital literacy, and improve wellbeing outcomes.

A key strength of this study is its structured, theory-informed development, incorporating findings from a behaviour analysis (Study 1) [19] and a co-design process with service users and staff (Study 2) [20]. This approach ensures that the intervention content, format, and delivery are tailored to the unique needs, capabilities, opportunities, and motivations of this population. Another strength is the mixed-method design, which enables quantitative assessment of dietary behaviours, digital literacy, and wellbeing; alongside qualitative exploration of participants’ experiences, perceptions, and barriers to engagement with the MindBia application.

Digital interventions provide scalable, flexible, and cost-effective opportunities to support lifestyle behaviour change [5,14], particularly for this population, who often face cognitive, motivational, and structural barriers to traditional interventions [28,29]. By embedding BCTs and monitoring engagement through in-app analytics, MindBia facilitates ongoing feedback, and structured support to help promote sustainable behaviour change.

There are several limitations to consider. As a pilot feasibility study, the sample size may be relatively small, and findings may not be generalisable to all individuals with SMI or other mental health settings. While digital interventions reduce barriers, participant engagement may still be influenced by digital literacy, motivation, or preferences for human contact and brief weekly check-ins may not fully address these factors for all users.

Despite these limitations, the study will provide valuable insights into the acceptability and practical delivery of a digital nutrition intervention for individuals with SMI. The findings are expected to inform refinements to the MindBia web-based application and guide the design of a larger-scale trial to evaluate effectiveness. Ultimately, the pilot study lays the groundwork for a scalable, person-centred, co-designed digital nutrition intervention that could complement traditional care and contribute to reducing the health disparities in the population.

## Supporting information

S1 Fig World Health Organisation – Five well-being index (WHO-5). (PNG)

S1 Text Semi-structured interview guide. (DOCX)

S2 Text Mediterranean diet adherence screener (MEDAS). (DOCX)

S3 Text Module-specific self-reported questionnaire. (DOCX)

S4 Text Adapted technology use survey. (DOCX)

## References

[1] Firth J, Siddiqi N, Koyanagi A, Siskind D, Rosenbaum S, Galletly C, et al. The Lancet Psychiatry Commission: a blueprint for protecting physical health in people with mental illness. Lancet Psychiatry. 2019;6(8):675–712. doi: 10.1016/S2215-0366(19)30132-4 31324560

[2] Halstead S, Cao C, Høgnason Mohr G, Ebdrup BH, Pillinger T, McCutcheon RA, et al. Prevalence of multimorbidity in people with and without severe mental illness: a systematic review and meta-analysis. Lancet Psychiatry. 2024;11(6):431–42. doi: 10.1016/S2215-0366(24)00091-9 38642560

[3] O’Connor RC, Worthman CM, Abanga M, Athanassopoulou N, Boyce N, Chan LF, et al. Gone Too Soon: priorities for action to prevent premature mortality associated with mental illness and mental distress. Lancet Psychiatry. 2023;10(6):452–64. doi: 10.1016/S2215-0366(23)00058-5 37182526

[4] Teasdale SB, Machaczek KK, Marx W, Eaton M, Chapman J, Milton A, et al. Implementing lifestyle interventions in mental health care: third report of the Lancet Psychiatry Physical Health Commission. Lancet Psychiatry. 2025;12(9):700–22. doi: 10.1016/S2215-0366(25)00170-1 40812962

[5] Brinsley J, O’Connor EJ, Singh B, McKeon G, Curtis R, Ferguson T. Effectiveness of digital lifestyle interventions on depression, anxiety, stress, and well-being: systematic review and meta-analysis. J Med Internet Res. 2025;27:e56975. doi: 10.2196/56975 PMC11969127 40112295

[6] Barber S, Thornicroft G. Reducing the Mortality Gap in People With Severe Mental Disorders: The Role of Lifestyle Psychosocial Interventions. Front Psychiatry. 2018;9:463. doi: 10.3389/fpsyt.2018.00463 30323773 PMC6172296

[7] Batra S, Baker RA, Wang T, Forma F, DiBiasi F, Peters-Strickland T. Digital health technology for use in patients with serious mental illness: a systematic review of the literature. Med Devices (Auckl). 2017;10:237–51. doi: 10.2147/MDER.S144158 29042823 PMC5633292

[8] Forde SA, Coppinger T, Rea S, Hanrahan S. The effectiveness of digital tools in physical activity interventions for individuals with severe mental illness: a scoping review. Disabil Rehabil Assist Technol. 2025;20(8):2594–615. doi: 10.1080/17483107.2025.2508938 40450697

[9] Torous J, Bucci S, Bell IH, Kessing LV, Faurholt-Jepsen M, Whelan P, et al. The growing field of digital psychiatry: current evidence and the future of apps, social media, chatbots, and virtual reality. World Psychiatry. 2021;20(3):318–35. doi: 10.1002/wps.20883 34505369 PMC8429349

[10] Hallberg D, Salimi N. Qualitative and Quantitative Analysis of Definitions of e-Health and m-Health. Healthc Inform Res. 2020;26(2):119–28. doi: 10.4258/hir.2020.26.2.119 32547809 PMC7278507

[11] Stefanopoulou E, Lewis D, Taylor M, Broscombe J, Larkin J. Digitally Delivered Psychological Interventions for Anxiety Disorders: a Comprehensive Review. Psychiatr Q. 2019;90(1):197–215. doi: 10.1007/s11126-018-9620-5 30488330

[12] Weightman M. Digital psychotherapy as an effective and timely treatment option for depression and anxiety disorders: Implications for rural and remote practice. J Int Med Res. 2020;48(6):300060520928686. doi: 10.1177/0300060520928686 32527170 PMC7294488

[13] Bond RR, Mulvenna MD, Potts C, O’Neill S, Ennis E, Torous J. Digital transformation of mental health services. Npj Ment Health Res. 2023;2(1):13. doi: 10.1038/s44184-023-00033-y 38609479 PMC10955947

[14] Sawyer C, Carney R, Hassan L, Bucci S, Sainsbury J, Lovell K, et al. Digital Lifestyle Interventions for Young People With Mental Illness: A Qualitative Study Among Mental Health Care Professionals. JMIR Hum Factors. 2024;11:e53406. doi: 10.2196/53406 38837191 PMC11187511

[15] O’Sullivan C, Merrotsy A, Coppinger T. The Role of Digital Nutrition Interventions for Individuals with Severe Mental Illness: Insights, Challenges, and Future Directions. Proc Nutr Soc. 2025;:1–28. doi: 10.1017/S0029665125102048 41424064

[16] Spanakis P, Lorimer B, Newbronner E, Wadman R, Crosland S, Gilbody S, et al. Digital health literacy and digital engagement for people with severe mental ill health across the course of the COVID-19 pandemic in England. BMC Med Inform Decis Mak. 2023;23(1):193. doi: 10.1186/s12911-023-02299-w 37752460 PMC10523616

[17] Michie S, van Stralen MM, West R. The behaviour change wheel: a new method for characterising and designing behaviour change interventions. Implement Sci. 2011;6:42. doi: 10.1186/1748-5908-6-42 21513547 PMC3096582

[18] Michie S, Atkins L, West R. The behaviour change wheel. A guide to designing interventions. 1st ed. Great Britain: Silverback Publishing. 2014.

[19] O’Sullivan C, Merrotsy A, Dhanapala I, Coppinger T. A behaviour analysis of nutrition behaviours and technology use of individuals with severe mental illness. Appetite. 2026;:108454. doi: 10.1016/j.appet.2026.108454 41513072

[20] O’Sullivan C, Coppinger T, Dhanapala I, Merrotsy A. Co-designing a digital nutrition intervention for individuals with severe mental illness using the behaviour change wheel. Mental Health and Digital Technologies. 2025;2(4):468–78. doi: 10.1108/mhdt-03-2025-0018

[21] Teresi JA, Yu X, Stewart AL, Hays RD. Guidelines for Designing and Evaluating Feasibility Pilot Studies. Med Care. 2022;60(1):95–103. doi: 10.1097/MLR.0000000000001664 34812790 PMC8849521

[22] Martínez-González MA, García-Arellano A, Toledo E, Salas-Salvadó J, Buil-Cosiales P, Corella D, et al. A 14-item Mediterranean diet assessment tool and obesity indexes among high-risk subjects: the PREDIMED trial. PLoS One. 2012;7(8):e43134. doi: 10.1371/journal.pone.0043134 22905215 PMC3419206

[23] Dwyer B, Mikkelson J, Diaz-Pacheco V, Myrick KJ, Torous J. The Technology Use Survey: An Actionable Digital Literacy Assessment that Matches Clients to Training Resources. Community Ment Health J. 2026;62(1):1–14. doi: 10.1007/s10597-025-01510-8 40921981

[24] Topp CW, Østergaard SD, Søndergaard S, Bech P. The WHO-5 Well-Being Index: A Systematic Review of the Literature. Psychother Psychosom. 2015;84(3):167–76. doi: 10.1159/000376585 25831962

[25] Knowledge translation, dissemination, and impact: a practical guide for researchers. Research and Development, Health Service Executive: Health Service Executive. 2021.

[26] Michie S, Richardson M, Johnston M, Abraham C, Francis J, Hardeman W, et al. The behavior change technique taxonomy (v1) of 93 hierarchically clustered techniques: building an international consensus for the reporting of behavior change interventions. Ann Behav Med. 2013;46(1):81–95. doi: 10.1007/s12160-013-9486-6 23512568

[27] Braun V, Clarke V. Thematic analysis: a practical guide. SAGE Publications. 2022.

[28] Teasdale SB, Samaras K, Wade T, Jarman R, Ward PB. A review of the nutritional challenges experienced by people living with severe mental illness: a role for dietitians in addressing physical health gaps. J Hum Nutr Diet. 2017;30(5):545–53. doi: 10.1111/jhn.12473 28419586

[29] Deenik J, Tenback DE, Tak ECPM, Blanson Henkemans OA, Rosenbaum S, Hendriksen IJM, et al. Implementation barriers and facilitators of an integrated multidisciplinary lifestyle enhancing treatment for inpatients with severe mental illness: the MULTI study IV. BMC Health Serv Res. 2019;19(1):740. doi: 10.1186/s12913-019-4608-x 31640706 PMC6806487

