# Supplementary material for: A pre-post pilot feasibility study of the MindBia web-based nutrition intervention for individuals with severe mental illness: Study protocol
Source: PLOS Digit Health. 2026 Sep 25;5(9):e0001756. doi: 10.1371/journal.pdig.0001756 (PMC13614651; doi:10.1371/journal.pdig.0001756)
Supplement: Supplementary file 1 [file pdig.0001756.s003.docx]

S2 Text. MEDAS

Mediterranean Diet Adherence Screener (MEDAS)

Adapted from: www.predimed.es, Int J Epidemiol. 2012 Apr; 41(2):377-385, J Nutr. 2011 Jun; 141(6):1140-1145 (Nov 2017)

		ANSWER	POINTS
1.	Do you use olive oil as the main source of fat for cooking?	Yes No	
2.	How many tablespoons of olive oil do you use each day?<br>*Include olive oil used in salads, meals eaten away from home, frying etc*	# tablespoons **per day**	
3.	How many servings of vegetables do you eat per day?<br>*One serving is ½ cup raw or cooked vegetables or 1 cup of raw salad greens*	# servings **per day**	
4.	How many servings of whole fruit do you eat per day?<br>*One serving is ½ cup or a medium sized piece of whole fruit*	# serving **per day**	
5.	How many servings of red meat, hamburger or sausages do you eat per week?<br>*One serving is 3 ½ -5 ½ ounces (100-150 grams)*	# servings **per week**	
6.	How many servings of butter, margarine or cream do you consume per day?<br>*One serving is 1 Tablespoon. This does not include soft non-hydrogenated margarines*	# servings **per day**	
7.	How many sugar sweetened beverages do you drink per week?<br>*One serving is 355ml or one can of pop or 12 ounces.*<br>*This includes any drinks with added sugars such as regular pop, fruit drinks, sports drinks, energy drinks, iced tea*	# servings **per week**	
8.	Do you drink wine? How much do you drink per week?<br>*1 glass = 150ml or 5 oz* **If you do not drink wine or alcohol, do not start**	# glasses **per week**	
9.	How many servings of legumes like kidney beans, chick peas, lentils, black beans, split peas do you eat per week?<br>*One serving is 5 ounces or 150 grams or ½ - 2/3 cup*	# servings **per week**	
10.	How many servings of fish or seafood do you eat per week?<br>*One serving of fish is 3 ½ -5 ½ ounces or 100-150 grams*<br>*One serving of seafood is 4-5 pieces or 7 ounces or 200 grams*	# servings **per week**	
11.	How many times do you eat baked goods such as pie, cookies, cake or doughnuts per week?	# times **per week**	
12.	How many times do you eat nuts per week?<br>*1 serving is 30 grams or 1 ounce*	# times **per week**	
13.	Do you eat chicken or turkey more often than beef, pork, hamburger or sausage?	Yes No	
14.	How many times per week do you eat dishes with a sauce of tomato, garlic, onion/leeks sautéed in olive oil?	# times **per week**	
		**TOTAL Points**	
