# Supplementary material for: A pre-post pilot feasibility study of the MindBia web-based nutrition intervention for individuals with severe mental illness: Study protocol
Source: PLOS Digit Health. 2026 Sep 25;5(9):e0001756. doi: 10.1371/journal.pdig.0001756 (PMC13614651; doi:10.1371/journal.pdig.0001756)
Supplement: Supplementary file 1 [file pdig.0001756.s002.docx]

**Interview Guide for Pre-Post Feasibility Study of the MindBia Web-Based Application for Individuals with Severe Mental Illness: A Pilot Study**

**Purpose of the Interview**

These semi-structured interviews are conducted post-intervention (week 8) to explore participants’ experiences of using the MindBia application, including perceived impacts on nutrition-related behaviours and digital literacy, as well as views on feasibility, acceptability, and sustainability of the intervention.

The interview guide has been developed based on the research aims and existing literature and will be piloted with co-design participants and refined accordingly.

**Interview Approach**

This interview will follow a semi-structured format. While a core set of questions will be used for all participants, additional follow-up questions may be asked depending on the direction of the conversation. Relevant topics that arise during earlier interviews may also be explored in subsequent interviews.

To support engagement and facilitate discussion, visual or interactive prompts may be used where appropriate (e.g. images or prompt cards illustrating food groups, shopping choices, and hydration behaviours).

**Guide**

**Introduction**

Thank you for taking part in this interview. This interview will take approximately 20-30 minutes. Your responses will be kept confidential, and you may skip any question or stop at any time.

**Questions**

**Overall Experience**

- What was your overall experience of using MindBia?
- What did you like the most?
- What did you like the least?

**Usability**

- Did you find the app easy to use?
- Were there any parts that were confusing or difficult?

**Feasibility and Acceptability**

- Was it easy to use the MindBia app each week?
- What made it easier to use?
- What made it harder to use?

**Nutrition Behaviours**

- Did you notice any changes in what or how you eat?
- Did anything from the app influence your food choices?

**Digital Confidence**

- Did your confidence using technology change?
- Do you feel more comfortable using apps now?

**Improvements and suggestions**

- What would you change about MindBia?
- What could make it better?

Is there anything else you would like to add?
