# Supplementary material for: A pre-post pilot feasibility study of the MindBia web-based nutrition intervention for individuals with severe mental illness: Study protocol
Source: PLOS Digit Health. 2026 Sep 25;5(9):e0001756. doi: 10.1371/journal.pdig.0001756 (PMC13614651; doi:10.1371/journal.pdig.0001756)
Supplement: Supplementary file 3 [file pdig.0001756.s004.docx]

**Module 2. Diet and Mental Health**

Q1. In a typical day, how many servings of dairy foods (e.g. milk , yogurt , cheese) do you usually consume?

☐ None
☐ 1 serving
☐ 2 servings

☐ 3 servings
☐ 4 or more servings

Q2. In the past 7 days, how many times did you eat takeaway or fast food?

☐ None
☐ 1-2 times
☐ 3-4 times
☐ 5 or more times

Q3. In the past 7 days, how many standard drinks did you have in total (any type)?

☐ 0 standard drinks
☐ 1 standard drink (half a pint of beer, stout or cider, small glass of wine, single measure of spirits)
☐ 2 standard drinks (a pint of beer, stout or cider, a double measure of spirits, a large can of beer)
☐ 3-6 standard drinks

☐ 7-11 standard drinks

☐ 12-17 standard drinks

☐ more than 17 standard drinks

**Module 3. Food Groups (Irish Food Pyramid)**

Q.1 How confident are you in your understanding of what a balanced meal looks like according to the Irish Food Pyramid?

☐ Not at all

☐ A little

☐ Somewhat

☐ Very

Q2. Do you know how many portions of fruit and vegetables you should eat per day?

☐ Yes

☐ No

☐ Not sure

b) if yes how many _________

**Q3.** How confident do you feel about knowing appropriate portion sizes for different food groups (e.g. wholemeal cereals and breads, potatoes, pasta and rice)

☐ Not at all

☐ A little

☐Somewhat

☐ Very

**Module 4. Meal Prep Skills**

Q1. In the past 7 days, how often have you prepared a meal by yourself?

☐ Never

☐ 1-2 days

☐ 3-4 days

☐ 5-7 days

Q2. How confident do you feel preparing a simple, healthy meal?

☐ Not at all

☐ A little

☐ Somewhat

☐ Very

**Module 5. Shopping**

Q1. In the past 7 days, how often did you buy fruits or vegetables when shopping for food for yourself?

☐ Never

☐ Rarely

☐ Sometimes

☐ Often

Q2. How confident do you feel in making healthy choices on a budget?

☐ Not at all

☐ A little

☐Somewhat

☐ Very

**Module 6. Hydration**

Q.1 In the past 7 days, how many days did you had a sugary drink? (not including fruit juice)

☐ 1-2 days

☐ 3-4 days

☐ 5 or more days

Q2. On a typical day in the past week, how many glasses of water did you drink?

☐ 0-1

☐ 2-3

☐ 4-5

☐ 6+

Q.3 How many cups of tea/coffee do you have a day?

☐ 0-1

☐ 2-3

☐ 4-5

☐ 6+
