# Supplementary material for: A pre-post pilot feasibility study of the MindBia web-based nutrition intervention for individuals with severe mental illness: Study protocol
Source: PLOS Digit Health. 2026 Sep 25;5(9):e0001756. doi: 10.1371/journal.pdig.0001756 (PMC13614651; doi:10.1371/journal.pdig.0001756)
Supplement: Supplementary file 5 [file pdig.0001756.s007.docx]

We sincerely thank the reviewers for their thoughtful and constructive feedback on our protocol manuscript. We have carefully considered all comments and revised the manuscript accordingly. We believe these revisions have strengthened the manuscript by improving its clarity and transparency. A detailed point by point is provided below.

| **Reviewer 1 Comments** | **Response** | **Reviewer 2 Comments** | **Response** |
| --- | --- | --- | --- |
| 1. The manuscript would benefit from clearer alignment with reporting guidelines, e.g., SPIRIT/CONSORT pilot/feasibility extensions, to better structure the methods and outcomes. | Thank you for this suggestion. The manuscript has been revised to improve the structure and reporting of the methods and outcomes, including clearer presentation of the study design, primary and secondary outcomes, recruitment procedures, setting, and statistical analysis. These revisions improve the overall transparency and reporting of the study protocol. | -Consider defining eHealth at it's first use and how this is different to 'digital interventions' named elsewhere. i.e.what is eHealth, mHealth, etc? This would increase the reach of this article through improved accessibility. | We have added a brief definition of electronic health (eHealth) at its first mention and clarified that mobile health (mHealth) is a subset of eHealth to improve clarity and accessibility for readers:  A diverse range of digital tools are increasingly integrated in mental health care (7–9). Electronic health (eHealth) refers to the use of information and communication technologies (ICT) to support health care delivery (10). Mobile health (mHealth), a subset of eHealth, and web-based platforms can deliver cost-effective, scalable, and flexible interventions that support healthier lifestyle behaviours (11,12). |
| 2. The primary outcomes of feasibility and acceptability could be more clearly stated. At present they become somewhat lost among the broader range of outcomes. In addition, these outcomes do not include predefined thresholds. It would be helpful to better frame them as the primary outcomes and add a priori criteria. | Thank you for this helpful suggestion. We agree that the primary feasibility and acceptability outcomes could be presented more clearly. The Outcome Measures section has been revised to distinguish **Primary Outcomes (Section 2.7.2)** from **Secondary Outcomes (Section 2.7.3)**, replacing the previous quantitative and qualitative outcome headings. We hope this revision more clearly identifies feasibility and acceptability as the primary outcomes of the pilot study, while nutrition-related behaviours, digital literacy, and general wellbeing are presented as secondary outcomes. | -why n=34? please justify. | ***Added to 2.4.1***  Participants will be recruited in collaboration with staff from high-support mental health hostels. As this is a pilot feasibility study, a formal sample size calculation was not undertaken. Consistent with recommendations for feasibility studies, the sample size was pragmatically based on the available study population (20). All eligible residents across the participating hostels (N=43) will be invited to participate through information sessions, study information leaflets, and staff referral |
| 3. Sample size is based on the number of people available across the sites. It would be helpful to contextualise this with reference to literature describing appropriate sample sizes for this type of study. | ***Added to 2.4.1***  As this is a pilot feasibility study, a formal sample size calculation was not undertaken. Consistent with recommendations for feasibility studies, the sample size was pragmatically based on the available study population (20). | -my understanding from reading is that phase 1 and 2 have been completed. in my opinion, have section 2.5.1 and 2.5.2 in this manuscript are somewhat confusing, as this protocol paper should be solely for the study to be undertaken. | We agree that the previous wording may have caused confusion regarding the scope of the current protocol. Sections 2.6.1 and 2.6.2 have been revised to clarify that Phases 1 and 2 were completed prior to the current pilot feasibility study and are included only to summarise the intervention development process that informed the MindBia application |
| 4. The analysis plan would benefit from additional detail regarding management of missing data, reporting of effect sizes, and how quantitative and qualitative findings will be integrated. | ***Added to 2.8***  Missing data will be reported descriptively, and analyses will be conducted using available data only. Effect sizes will be calculated, where appropriate, to estimate the magnitude of any observed changes.  ***Added to 2.8***  Quantitative and qualitative findings will be integrated at the interpretation stage to provide a comprehensive understanding of the feasibility, acceptability, and preliminary outcomes of the MindBia intervention. | -good analysis, stating this is not powered and will be a pilot study. would effect sizes and or % change be useful to include? | **Added to 2.8**  Missing data will be reported descriptively, and analyses will be conducted using available data only. Effect sizes will be calculated, where appropriate, to estimate the magnitude of any observed changes. |
| 5. The protocol would benefit from clearer description of how capacity to provide informed consent will be assessed, including whether this will be determined by clinician judgement or through a structured assessment (e.g., UBACC). Capacity is not synonymous with being in or out of an acute episode, and an explicit statement would improve transparency. | ***Added to 2.4.1***  Capacity to provide informed consent will be determined by the relevant clinician prior to study enrolment. | -as the platform is already developed, some screengrabs would inform the reader about the platform. the UI could be interesting to see. | **Added to 2.6.3**  Representative screenshots of the MindBia web-based application are provided in Supplementary File 1. |
| 6. The setting description would benefit from additional contextual details (e.g., hostel size range, level of support, staffing model, and geographical locations). | ***Added to 2.3***  The study will be conducted in four high-support mental health hostels located in the south-west of Ireland. These residential settings provide accommodation and 24-hour structured support for adults with severe mental illness. Daily support is provided by mental health nurses, healthcare assistants, and household staff, with clinical oversight provided by a consultant psychiatrist and dedicated doctor across the four hostels. The participating hostels vary in size, with capacity ranging from 8 to 14 residents. | -supplementary file 1 is cited but i cannot see it. | I have updated all supplementary files |
| 7. Where possible, additional details on the qualitative methods would be useful, including sampling, data collection, underlying stance and analytic approach. | ***Added to 2.7.2.2***  Acceptability will be explored through post-intervention semi-structured interviews (week 8) (Supplementary File 2) conducted with a purposive sample of participants.  ***Added to 2.7.2.2***  The qualitative component is informed by an interpretivist perspective, recognising that participants’ experiences and perceptions of the intervention may be shaped by their individual contexts.  ***Added to 2.8***  Qualitative interview will be audio-recorded, transcribed verbatim and analysed using reflexive thematic analysis (Braun and Clarke, 2022). | -would the MAUQ be a good outcome measure to include? <https://mhealth.jmir.org/2019/4/e11500/> | Thank you for this suggestion. We considered including the MAUQ. However, usability of the MindBia platform was previously evaluated during the co-design phase using the validated System Usability Scale (SUS), and the resulting feedback informed refinements to the intervention prior to this pilot study. |
| 8. The final paragraph of the introduction and section 2.1 overlap considerably. It may help to have section 2.1 to focus more specifically on study design rather than repeating the objectives and potentially include the reporting guideline informing your protocol. | ***Changed 2.2 to***  This protocol describes an eight-week mixed-method pre-post feasibility study of the MindBia web-based nutrition intervention. The study will incorporate quantitative outcome measures, app usage analytics, and post-intervention qualitative interviews to evaluate the implementation and delivery of the intervention. |  |  |
