# Supplementary material for: A pre-post pilot feasibility study of the MindBia web-based nutrition intervention for individuals with severe mental illness: Study protocol
Source: PLOS Digit Health. 2026 Sep 25;5(9):e0001756. doi: 10.1371/journal.pdig.0001756 (PMC13614651; doi:10.1371/journal.pdig.0001756)
Supplement: Supplementary file 7 [file pdig.0001756.s005.docx]

S4 Text. Technology Use Survey
