# Supplementary material for: A pre-post pilot feasibility study of the MindBia web-based nutrition intervention for individuals with severe mental illness: Study protocol
Source: PLOS Digit Health. 2026 Sep 25;5(9):e0001756. doi: 10.1371/journal.pdig.0001756 (PMC13614651; doi:10.1371/journal.pdig.0001756)
Supplement: Peer review history

# Peer review: Decision Letter 0

**DOI:** 10.1371/journal.pdig.0001756.r001

**Authors:** Dukyong Yoon, Nilihan Sanal-Hayes

6 Jul 2026

Response to Reviewers Revised Manuscript with Track Changes Manuscript **Journal Requirements:**

1. Please amend your detailed Financial Disclosure statement. This is published with the article. It must therefore be completed in full sentences and contain the exact wording you wish to be published.

i. Please clarify all sources of financial support for your study. List the grants, grant numbers, and organizations that funded your study, including funding received from your institution. Please note that suppliers of material support, including research materials, should be recognized in the Acknowledgements section rather than in the Financial Disclosure.

ii. State the initials, alongside each funding source, of each author to receive each grant. For example: "This work was supported by the National Institutes of Health (####### to AM; ###### to CJ) and the National Science Foundation (###### to AM)."

iii. State what role the funders took in the study. If the funders had no role in your study, please state: “The funders had no role in study design, data collection and analysis, decision to publish, or preparation of the manuscript.”

iv. If any authors received a salary from any of your funders, please state which authors and which funders.

2. Please provide a complete Data Availability Statement in the submission form, ensuring you include all necessary access information or a reason for why you are unable to make your data freely accessible. If your research concerns only data provided within your submission, please write "All data are in the manuscript and/or supporting information files" as your Data Availability Statement.

3. We note that you have included your Figures within the body of your manuscript. Please remove the Figures from the body of your manuscript and upload them as separate Figure files in .tif or .eps format.

For more information about figure files please see our guidelines:

https://journals.plos.org/digitalhealth/s/figures

https://journals.plos.org/digitalhealth/s/figures#loc-file-requirements

4. Please ensure that your Ethics Statement is available in its entirety at the beginning of your Methods section, under a subheading 'Ethics Statement'.

5. Please ensure all the standard sections present, (e.g. Abstract, Introduction, Results, Discussion, Methods), in the correct order, and that any references to subheadings in your main text are correct. An outline of the required sections can be consulted in our submission guidelines here:

https://journals.plos.org/digitalhealth/s/submission-guidelines#loc-parts-of-a-submission

**Additional Editor Comments (if provided):** **Reviewers' Comments:**

**Comments to the Author**

1. Does this manuscript meet PLOS Digital Health’s publication criteria?

Reviewer #1: Yes

Reviewer #2: Yes

**********

2. Has the statistical analysis been performed appropriately and rigorously?

Reviewer #1: N/A

Reviewer #2: Yes

**********

3. Have the authors made all data underlying the findings in their manuscript fully available (please refer to the Data Availability Statement at the start of the manuscript PDF file)?

The PLOS Data policy

Reviewer #1: Yes

Reviewer #2: Yes

**********

4. Is the manuscript presented in an intelligible fashion and written in standard English?

Reviewer #1: Yes

Reviewer #2: Yes

**********

**Reviewer #1:**  This is a well-written and timely protocol addressing an important gap in digital nutrition interventions for people with severe mental illness. The development process is a clear strength and I look forward to seeing the findings. I have outlined a couple of points to consider, along with some minor clarifications that would improve transparency and reproducibility.

1. The manuscript would benefit from clearer alignment with reporting guidelines, e.g., SPIRIT/CONSORT pilot/feasibility extensions, to better structure the methods and outcomes.

2. The primary outcomes of feasibility and acceptability could be more clearly stated. At present they become somewhat lost among the broader range of outcomes. In addition, these outcomes do not include predefined thresholds. It would be helpful to better frame them as the primary outcomes and add a priori criteria.

3. Sample size is based on the number of people available across the sites. It would be helpful to contextualise this with reference to literature describing appropriate sample sizes for this type of study.

4. The analysis plan would benefit from from additional detail regarding management of missing data, reporting of effect sizes, and how quantitative and qualitative findings will be integrated.

5. The protocol would benefit from clearer description of how capacity to provide informed consent will be assessed, including whether this will be determined by clinician judgement or through a structured assessment (e.g., UBACC). Capacity is not synonymous with being in or out of an acute episode, and an explicit statement would improve transparency.

6. The setting description would benefit from additional contextual details (e.g., hostel size range, level of support, staffing model, and geographical locations).

7. Where possible, additional details on the qualitative methods would be useful, including sampling, data collection, underlying stance and analytic approach.

8. The final paragraph of the introduction and section 2.1 overlap considerably. It may help to have section 2.1 to focus more specifically on study design rather than repeating the objectives, and potentially include the reporting guideline informing your protocol.

**Reviewer #2** : Thank you for submitting this manuscript. Please find some suggestions below:

-Consider defining eHealth at it's first use and how this is different to 'digital interventions' named elsewhere. i.e.what is eHealth, mHealth, etc? This would increase the reach of this article through improved accessibility.

-why n=34? please justify.

-my understanding from reading is that phase 1 and 2 have been completed. in my opinion, have section 2.5.1 and 2.5.2 in this manuscript are somewhat confusing, as this protocol paper should be solely for the study to be undertaken.

-good analysis, stating this is not powered and will be a pilot study. would effect sizes and or % change be useful to include?

-as the platform is already developed, some screengrabs would inform the reader about the platform. the UI could be interesting to see.

-supplementary file 1 is cited but i cannot see it.

-would the MAUQ be a good outcome measure to include? https://mhealth.jmir.org/2019/4/e11500/

**********

what does this mean?). If published, this will include your full peer review and any attached files.

**Do you want your identity to be public for this peer review?**  If you choose “no”, your identity will remain anonymous but your review may still be made public.

For information about this choice, including consent withdrawal, please see our Privacy Policy

Reviewer #1: **Yes:**  Scott B. Teasdale

Reviewer #2: **Yes:**  Dr Lawrence D. Hayes

**********

**Figure resubmission:**

**Reproducibility:**

To enhance the reproducibility of your results, we recommend that authors of applicable studies deposit laboratory protocols in protocols.io, where a protocol can be assigned its own identifier (DOI) such that it can be cited independently in the future. Additionally, PLOS ONE offers an option to publish peer-reviewed clinical study protocols. Read more information on sharing protocols at https://plos.org/protocols?utm_medium=editorial-email&utm_source=authorletters&utm_campaign=protocols

# Author comment: Author response to Decision Letter 1

**DOI:** 10.1371/journal.pdig.0001756.r002

14 Jul 2026

Attachment Submitted filename: Response to reviewers .docx

# Peer review: Decision Letter 1

**DOI:** 10.1371/journal.pdig.0001756.r003

**Authors:** Dukyong Yoon

7 Sep 2026

A Pre-Post Pilot Feasibility Study of the MindBia Web-Based Nutrition Intervention for Individuals with Severe Mental Illness: Study Protocol

PDIG-D-26-00451R1

Dear O'Sullivan,

We are pleased to inform you that your manuscript 'A Pre-Post Pilot Feasibility Study of the MindBia Web-Based Nutrition Intervention for Individuals with Severe Mental Illness: Study Protocol' has been provisionally accepted for publication in PLOS Digital Health.

Before your manuscript can be formally accepted you will need to complete some formatting changes, which you will receive in a follow-up email from a member of our team.

Please note that your manuscript will not be scheduled for publication until you have made the required changes, so a swift response is appreciated.

IMPORTANT: The editorial review process is now complete. PLOS will only permit corrections to spelling, formatting or significant scientific errors from this point onwards. Requests for major changes, or any which affect the scientific understanding of your work, will cause delays to the publication date of your manuscript.

If your institution or institutions have a press office, please notify them about your upcoming paper to help maximize its impact. If they'll be preparing press materials, please inform our press team as soon as possible -- no later than 48 hours after receiving the formal acceptance. Your manuscript will remain under strict press embargo until 2 pm Eastern Time on the date of publication. For more information, please contact digitalhealth@plos.org.

Thank you again for supporting Open Access publishing; we are looking forward to publishing your work in PLOS Digital Health.

Best regards,

Dukyong Yoon

Section Editor

PLOS Digital Health

***********************************************************

**Additional Editor Comments (if provided):**

**Reviewer Comments (if any, and for reference):**

Reviewer's Responses to Questions

**Comments to the Author**

Reviewer #1: All comments have been addressed

Reviewer #2: All comments have been addressed

**********

publication criteria?

Reviewer #1: Yes

Reviewer #2: Yes

**********

3. Has the statistical analysis been performed appropriately and rigorously?

Reviewer #1: N/A

Reviewer #2: Yes

**********

4. Have the authors made all data underlying the findings in their manuscript fully available (please refer to the Data Availability Statement at the start of the manuscript PDF file)?

The PLOS Data policy

Reviewer #1: No

Reviewer #2: Yes

**********

5. Is the manuscript presented in an intelligible fashion and written in standard English?

Reviewer #1: Yes

Reviewer #2: Yes

**********

Reviewer #1: Thank you for the revised manuscript. My comments have been addressed satisfactorily.

Reviewer #2: thank you for addressing suggestions

**********

what does this mean?). If published, this will include your full peer review and any attached files.

**Do you want your identity to be public for this peer review?**  If you choose “no”, your identity will remain anonymous but your review may still be made public.

For information about this choice, including consent withdrawal, please see our Privacy Policy

Reviewer #1: **Yes:**  Scott Teasdale

Reviewer #2: **Yes:**  Dr Lawrence Hayes

**********
